# Three Cases of Nasal Alimentation

**Published:** 1883-01-20

**Authors:** D. N. Kankin

**Affiliations:** Alleghany City, Pennsylvania


### THREE CASES OF NASAL ALIMENTATION.

By D. N. Kankin, A. M., M. D., of Alleghany City, Penn.

Case 1.—John McC., æt. 28 years, of an anæmic appearance, had, on Friday, Sep ember 19, 1879, a molar tooth of lower jaw extracted by a dentist; bleeding from the socket continued through Friday night, and all day Saturday. On Saturday evening I was sent for to stop it. Upon examination I found the blood oozing quite copiously from numerous points. I learned from some of his friends that the man was of a hemorrhagic diathesis; they informed me that he had almost bled to death under similar circumstances, on two previous occasions. I applied the usual styptics, with no benefit whatever; then the actual cautery was applied, with no better result. I then concluded to pack the cavity with lint, saturated with sol. persulphate of iron (Monsel's) with beeswax over it, and a cork compress over all; the lower jaw was firmly bound to the upper one by a bandage over the top of the head and under the chin. This plan succeeded in checking the hemorrhage, but to make it successful the appliance was retained several days. Now came the most trying part of the treatment: how was this man to be nourished? The idea at once presented itself to my mind to introduce nutritious fluids through the nares. I secured a sol. citrate of magnesia bottle, had the bottom filed off, put a small glass tube through the cork (which had previously been placed in the mouth of the bottle); to this a sufficiently small-sized gum tube, to pass through the nares easily, was attached; after oiling the tube I introduced it into one of the nares, past the pharynx, into the œsophagus; it worked admirably. At the first operation, one half pint of milk and one half pint of beef tea were introduced; this procedure was continued three times a day, for four days, until Wednesday, September 24th, when the cork and wax were removed, and no further hemorrhage occurred.

Case 2.—Tillie R., æt. 24 years, was admitted into the Western Penitentiary of Pennsylvania, in June, 1875, for a term of three years. At the time of her admission she was six months advanced in pregnancy. At her expected time she was delivered of a son; everything progressed favorably; for eighteen months she was fed on the best food the prison could afford, in order that she might supply nourishment of good quality and sufficient quantity for the child. When the babe was eighteen months old, I deemed it proper to wean it, and from this time she was ordered to eat the regular prison food. From that day the interesting part of this case commenced; she positively declared that unless she was supplied with the same kind of food she had been having since her child was born, she would take none, and said she would starve herself to death. This, I told her, she could not do; she defied me; a quart of beef tea was soon in readiness, and with the aid of four stout men, and with considerable difficulty, the quart of beef tea was introduced into her stomach by means of a stomach pump. Her persistency in still trying to starve was so great that I concluded to try some more easy, at the same time equally efficacious, means of introducing nutritious fluids into her stomach; a gum tube, two and a half feet long and one-fourth of an inch in diameter, was secured, and a small tin funnel fitted to one end of the tube. Everything being in readiness, she was again held by four men; this time she anticipated the procedure by holding her lips and teeth firmly, expecting that I was going to introduce the tube of the stomach-pump, as had been done previously. But before she was aware of it, the soft gum tube, well oiled, was passed through one of the nostrils, and the greater part of a pint of milk was introduced through it into the stomach. It was only then that she found all her efforts at keeping her lips and teeth so firmly closed did not baffle me in the effort I had undertaken. The same procedure was gone through with three times a day for three days. On the fourth day she concluded, as she remarked, to take her meals in the regular way.

CASE 3.—On the 21st of October, 1880, I was sent for to visit Mr. J. G.. æt. about 35 years, both physically and mentally a wreck. This being one of his attacks of mental depression, his friends stated that he had refused to eat or drink anything for the past two days, and that he had intended destroying his life by starvation, as he said he was of no further use in this world. Upon my arrival I found the man in a very melancholy condition, and it was with a great deal of difficulty that an answer to my questions could be obtained from him. When I interrogated him in regard to taking his food, he remarked that his mind was already made up upon that point; that nothing more should pass his mouth! All my appeals being of no avail, I determined the man should not die if it was in my power to prevent it. His friends informed me that this was not the first time he attempted to starve himself, but that some two years ago, when living in a Western city, he was in about the condition as at present, and the doctors in attendance upon him made an effort to use the stomach-pump, but with his stong teeth he crushed the gag and cut the tube through The same gum tube and funnel that was used in Case No. 2 was utilized for this patient. Everything being in readiness, with four stout men to secure him (I presume, from the manner in which he held his lips and teeth, he supposed the stomach-pump was going to be used again, and in this supposition I encouraged him) before he was aware of it, the gum tube had been passed through one of the nostrils and œsophagus, and a pint of beef tea introduced into the stomach. So persistent was he to destroy his life in this manner, that this same procedure had to be repeated three times a day for one week, until finally he concluded to take his food in the usual way. It is proper to state here that positive directions were given the attendants in charge of the two latter cases, to watch them carefully for at least two hours after the operation, in order to prevent them inducing vomiting by titillating the upper part of the œsophagus with the finger.—(*Archives of Laryngology, October 1882.*)—*Clin. Rec.*

